# Supplementary material for: Media consumption and psychological distress among older adults in the United States
Source: PLoS One. 2022 Dec 30;17(12):e0279587. doi: 10.1371/journal.pone.0279587 (PMC9803103; doi:10.1371/journal.pone.0279587)
Supplement: S1 Appendix — (DOCX) [file pone.0279587.s001.docx]

**Appendix**

Table A1 provides descriptive statistics and the origins of the measures in our scale of psychological distress.

| **Table A1. Origins and descriptive statistics for the measures included in the psychological distress scale.** | | | |
| --- | --- | --- | --- |
| Source | Item | Mean | Std Dev |
| GAD-7 | Felt nervous, anxious, or on edge | 2.10 | 1.02 |
| CESD-20 | Felt depressed | 1.61 | 0.85 |
| CESD-20 | Felt lonely | 1.49 | 0.80 |
| CESD-20 | Felt hopeful about the future | 2.20 | 1.01 |
| CESD-20 | Had trouble sleeping | 1.89 | 0.99 |
| *Notes*: Weighted descriptive statistics. | | | |

Tables A2 and A3 provide estimates that are illustrated in Figure 1 and Figure 2, Panel A.

| **Table A2. Estimates from regressing psychological distress on media consumption and other covariates; N = 2,781.** | |
| --- | --- |
|  | Est (SE) |
| Very close media consumption | 0.63 (0.13)*** |
| Non-Hispanic Black | -0.42 (0.35) |
| Non-Hispanic White | -0.72 (0.28)** |
| Female | 0.64 (0.12)*** |
| Some college | -0.48 (0.15)*** |
| College degree or higher | -0.26 (0.14) |
| Married or cohabiting | -0.50 (0.12)*** |
| Live in metro area | 0.18 (0.17) |
| Midwest | 0.04 (0.19) |
| South | -0.06 (0.18) |
| West | 0.05 (0.19) |
| Prior mental health condition | 2.50 (0.21)*** |
| Intercept | 9.33 (0.38)*** |
| *Notes*: *p < 0.05, **p < 0.01, *** p < 0.001. Unstandardized estimates with standard errors in parentheses. Model incorporates sample weights. | |

| **Table A3. Estimates for very close media consumption from regressing psychological distress on media consumption and other covariates for indicated subsamples.** | | |
| --- | --- | --- |
| Subsample | N | VC Est (SE) |
| Women | 1,349 | 0.85 (0.20)*** |
| Men | 1,432 | 0.47 (0.17)*** |
| Hispanic | 299 | 2.33 (0.44)*** |
| non-Hispanic Black | 140 | 1.32 (0.60)* |
| non-Hispanic White | 2,342 | 0.56 (0.14)*** |
| College degree or higher | 1,572 | 1.05 (0.18)*** |
| Some college | 842 | 0.70 (0.25)** |
| High school | 367 | 0.41 (0.35) |
| Married/cohabiting | 1,813 | 0.73 (0.16)*** |
| Not married/cohabiting | 968 | 0.60 (0.23)** |
| *Notes*: *p < 0.05, **p < 0.01, *** p < 0.001. Unstandardized estimates for very close media consumption with standard errors in parentheses. Model incorporates sample weights. | | |
